# Efficacy and tolerability of native (undenatured) type II collagen supplementation for joint health in healthy volunteers: a randomized double-blind placebo-controlled study

**DOI:** 10.1186/s12937-026-01302-0

**Published:** 2026-03-06

**Authors:** Ingrid Möller, Kirsten Martínez, Andrea Terradillos-Guillén, Ester Costa-Larrión, Daniel Martínez-Puig, Javier Velasco-Álvarez

**Affiliations:** 1 Instituto Poal de Reumatología, Barcelona, Spain; 2 R&D Bioiberica S.A.U. 08389, Palafolls (Barcelona), Spain

**Keywords:** Native type-II collagen, Undenatured type-II collagen, Joint discomfort, Knee joint, KOOS, CTX-II

## Abstract

**Background:**

In non-osteoarthritic individuals, the appearance of joint discomfort with physical activity is a potential indicator of initial joint degeneration. The present study aimed to evaluate the efficacy and tolerability of native (undenatured) type-II collagen in the joint function of healthy volunteers who experienced joint discomfort as a result of physical activity.

**Methods:**

This prospective, randomized, double-blind, placebo-controlled-study, included seventy-four healthy subjects with joint discomfort during physical activity. Participants were randomized to receive placebo (PBO) or native (undenatured) type-II collagen (CN2) at 40 mg/d for 180 days. Joint function and time to recover from knee discomfort after a standardized exercise protocol were evaluated. CTX-II was analyzed as biomarker of cartilage degradation.

**Results:**

In both groups a significant improvement in joint function measured with the KOOS score was detected at 180 days, but improvements from baseline were detected earlier in the CN2 as compared to PBO group for KOOS pain, Symptoms and Quality of Life. In the subgroups of subjects with milder basal joint discomfort during activity (VAS≤40 mm) differences between groups in improvement from baseline were detected at 180 days for KOOS pain and Quality of Life subscales. Between-group differences were also detected in time to recover after cycling in the air test. In the subgroups of subjects with higher basal joint discomfort during activity (VAS>40 mm) between-group differences were detected on the progression of CTX-II concentration from baseline, which decreased in CN2 and increased in PBO group.

**Conclusions:**

This study found that supplementation with CN2, containing native (undenatured) type II collagen supports certain aspects of joint health in people who experience joint discomfort after exercise, including improvements in joint function, discomfort and their impact on quality of life.

**Trial registration:**

The study was registered at ClinicalTrials.gov (NCT05282992) on March 16, 2022.

**Supplementary Information:**

The online version contains supplementary material available at 10.1186/s12937-026-01302-0.

## Background

Osteoarthritis (OA) is the most common joint disorder affecting up to 14.8% of the global population older than 30 years [1]. In 2020, OA was a top-ten leading cause of years lived with disability (YLD), and its prevalence is expected to rise as a result of the increase in life expectancy, becoming a major socioeconomic and public health issue [1–3].

The therapeutic approach for OA has been focused on reducing pain and improving physical function. Pharmacological interventions are mainly based on analgesics (acetaminophen) and non-steroidal anti-inflammatory drugs (NSAIDS) [4], but safety concerns could restrict their long-term use, particularly in patients with comorbidities [5, 6]. Despite significant efforts in research during the last decades, there are no approved drugs to modify the progression of joint structural damage [7, 8]. Consequently, when patients are diagnosed according to clinical and imaging examination, often irreversible damage has occurred [9]. This context has expanded the focus of research towards the risk factors of OA [10]. While therapeutic exercise has been shown to provide benefits in people with OA [11], a high daily dose of loading exercise could promote cartilage damage [12, 13]. In fact, mechanical joint loading has been used as a model to study OA initiation and progression [14–16]. Joint loading has been associated with synovial inflammation [17], which has been suggested to play a role in the onset and structural progression of OA [18]. Although there is no clear consensus on the correlation between the severity of joint structural degradation and pain severity [19, 20], a positive correlation has been described with the joint loading model [13].

Supplementation with native (undenatured) type-II collagen has been shown to help to control inflammation at the joint level through an immune-mediated mechanism of oral tolerance [21, 22]. Collavant^®^ n2 (CN2) is a natural ingredient derived from chicken cartilage that contains native (undenatured) type II collagen. In a rat model of OA induced by monoiodoacetate (MIA), the oral administration of CN2 reduced the concentration of inflammatory cytokines (TNFα, IL-1β) and decreased cartilage degradation [23]. The reduction of cartilage degradation correlated with a reduction on nociceptive behaviour. A clinical trial in patients with OA, showed that the concomitant administration of CN2 and acetaminophen over 3 months improved pain during walking to a greater extent than acetaminophen alone [24].

Although the mechanism of oral tolerance has been defined for OA and rheumatoid arthritis (RA) [21], and exercise-related joint discomfort is not considered a risk factor of early osteoarthritis [25], we used the appearance of discomfort during physical activity as an exploratory model of joint loading stress to evaluate the potential impact of CN2 supplementation on the evolution of different joint health associated outcomes in healthy population.

## Methods

### Participants

The study included healthy subjects reaching a joint discomfort of 5/10 on a 11-point Likert scale (scale of 0 to 10) within 10 min of going up and down stairs and pain score assessed with a Visual Analogue Scale (VAS) during knee movement between 30 and 50 mm (scale of 0–100 mm). All participants met the eligibility criteria described in Table 1. All randomized subjects continued the study procedures and attended the scheduled follow-up visits until the study ended, unless they voluntarily abandoned the study or failed to comply with the study protocol.

**Table 1:** Eligibility criteria

Inclusion	Exclusion
• Males and non-pregnant females 30–65 years old with a BMI) 18 to 30 kg/m2. • Unilateral/bilateral knee discomfort > 3 months. • VAS score during knee movement 30–50 mm.• Joint discomfort of 5/10 on an 11-point Likert scale (scale of 0 to 10) within 10 min of an going up and down stairs. • Clinical laboratory results within normal range. • Be willing to participate in all tests in the protocol. • Be willing to refrain from taking pain reliever during the entire study other than acetaminophen (paracetamol) as rescue medication. • Provide a signed and dated informed consent. • Be willing to refrain from taking dietary supplements that have any underlying joint benefit.	• History of hypersensitivity to rescue medication or to the supplement. • Requirement of drugs to control joint discomfort. • History of Knee OA, inflammatory arthropathy, RA, OA (VAS score greater than 50), or Systemic Lupus Erythematosus. Past or recent injury in the target knee. Evidence of clinically significant diseases or malignancies within the last 5 years. • Exercising for more than 10 h a week. • Pregnant or lactating females • High alcohol intake (> 2 standard drinks per day) or drugs • Use of oral corticosteroids, indomethacin, SYSADOAs within 3 months of Visit 1; topical treatment with corticosteroids within 1 week of visit 1, and consumption of Omega 3 fatty acids or any other joint health dietary supplements within 2 weeks preceding the treatment period. • Consumed, ibuprofen, aspirin or other NSAIDS, or any other pain reliever (OTC or prescription), or any natural health product, (excluding vitamins) within 7 days of first visit. • Consumed acetaminophen (paracetamol) within 48 h of randomization visit. • Participation in any clinical trials within 30 days prior to first visit.

### Interventions

The investigational product, 40 mg of CN2 per day, was provided by Bioiberica SAU. Placebo (PBO) capsule was sensory identical and contained only excipients (microcrystalline cellulose, magnesium stearate). The labelling of packages complied with the regulatory requirements of Spain as well as the recommendations in Appendix 13 of the European Guide to Good Manufacturing Practice (European Commission, 2010). Subjects were instructed to take one capsule on an empty stomach 30 min before breakfast. Investigational products accountability was under the responsibility of the investigator. Treatment management was verified on a regular basis by the study monitor. Product intake was assessed monthly, either at the scheduled visits or by a follow-up phone call.

### Study design

This 6-month randomized, double-blind, placebo-controlled, parallel study with an allocation ratio 1:1, was conducted at Instituto Poal de Reumatología in Barcelona, Spain. All participants recruited were informed about the objectives, protocol, risks, and benefits before voluntarily giving written consent. Eligible participants were randomly allocated to CN2 or PBO by centralized randomization at the inclusion visit (1:1). The randomization was performed by an independent statistician not involved in conducting the study using a sequential block randomization list with a block size of ten. To guarantee double blinding, CN2 and PBO were provided in the form of capsules with identical appearance. All participants and investigators remained blinded to the treatment allocation through the trial.

### Efficacy assessments

The present study aimed to determine the differences from baseline and between groups on joint discomfort onset, defined as the primary outcome, and on recovery from joint discomfort caused by activity and evolution of knee function and discomfort, all of them defined as secondary outcomes. Included participants were required to undergo a series of standardized exercise protocols including going up and down stairs (UDS), standing in one leg (SOL) and cycling in the air (CIA) at baseline and at 60-, 90-, 120- and 180-days. Time to onset and recovery of knee discomfort were measured, as well as the Visual Analogue Scale (VAS) and the 6 min walking test. Efficacy was also assessed at each visit as per the Knee injury and Osteoarthritis Outcome Score (KOOS). The KOOS score is a validated instrument consisting of 42 questions that are classified into sub-scales such as Pain, other Symptoms, Function in daily living (ADL), Function in Sport and Recreation (Sport/Rec) and knee-related Quality of Life (QOL). It measures the subjects’ opinion about their knees and their ability to perform daily activities during the past week. A change of 10 points or more has been used as cutoff value representing a clinically significant difference [26]. Urinary samples were obtained at baseline and at the end of the study. CTX-II concentration, defined as exploratory variable, was standardized to the total urine creatinine at the time of sampling. CTX-II concentrations were determined using an enzyme immunoassay (EIA) kit (Urine CartiLaps^®^ EIA; Immunodiagnostic systems, Boldon, UK).

### Safety assessments

Physical examination, abnormal parameters, weight, BMI, SBP, DBP. Heart rate, consumption of rescue medication, intake of new medications and adverse events since the last visit were registered to assess the safety and tolerance of the intervention.

### Statistical analysis

The final sample size was set at 100 subjects but a minimum of 66 participants were needed to reach statistical results taking into account potential dropouts or other protocol deviations. No interim analysis of efficacy was planned for this study. A subgroup analysis was preplanned of the subjects with baseline VAS score≤4 cm, defined as a cutoff value to distinguish mild from moderate intensity [27]. All analysis and data management were performed using SAS System^®^ for Windows, release 9.4 or later (SAS^®^ Institute Inc., Cary, NC, USA) at the 5% two-sided level. No multiplicity corrections were performed. Efficacy endpoints were evaluated by means of a T-test. The evaluation of the application criteria of the parametric tests was carried out using the Shapiro-Wilk and Levene tests to evaluate normality and homogeneity of variances respectively. The analysis of the results was carried out in the mITT population, defined as all those participants who were randomized and received the study interventions. Missing data were imputed by the last observation carried forward approach.

### Ethical aspects

The study was conducted following the guidelines for Good Clinical Practice and following the Declaration of Helsinki (E8) for treatment of human subjects in a study. This study was approved by the local ethics committee IDIAP Jordi Gol Ethics Committee (CEIC code: 21/243- ACpn) and the clinical trial was registered at ClinicalTrials.gov with code NCT05282992 on March 16, 2022. The study was conducted and reported according to the Consolidated Standards of Reporting Trials (CONSORT) guidelines (CONSORT checklist in Supplementary File S1).

## Results

### Participants characteristics

**Fig. 1:**
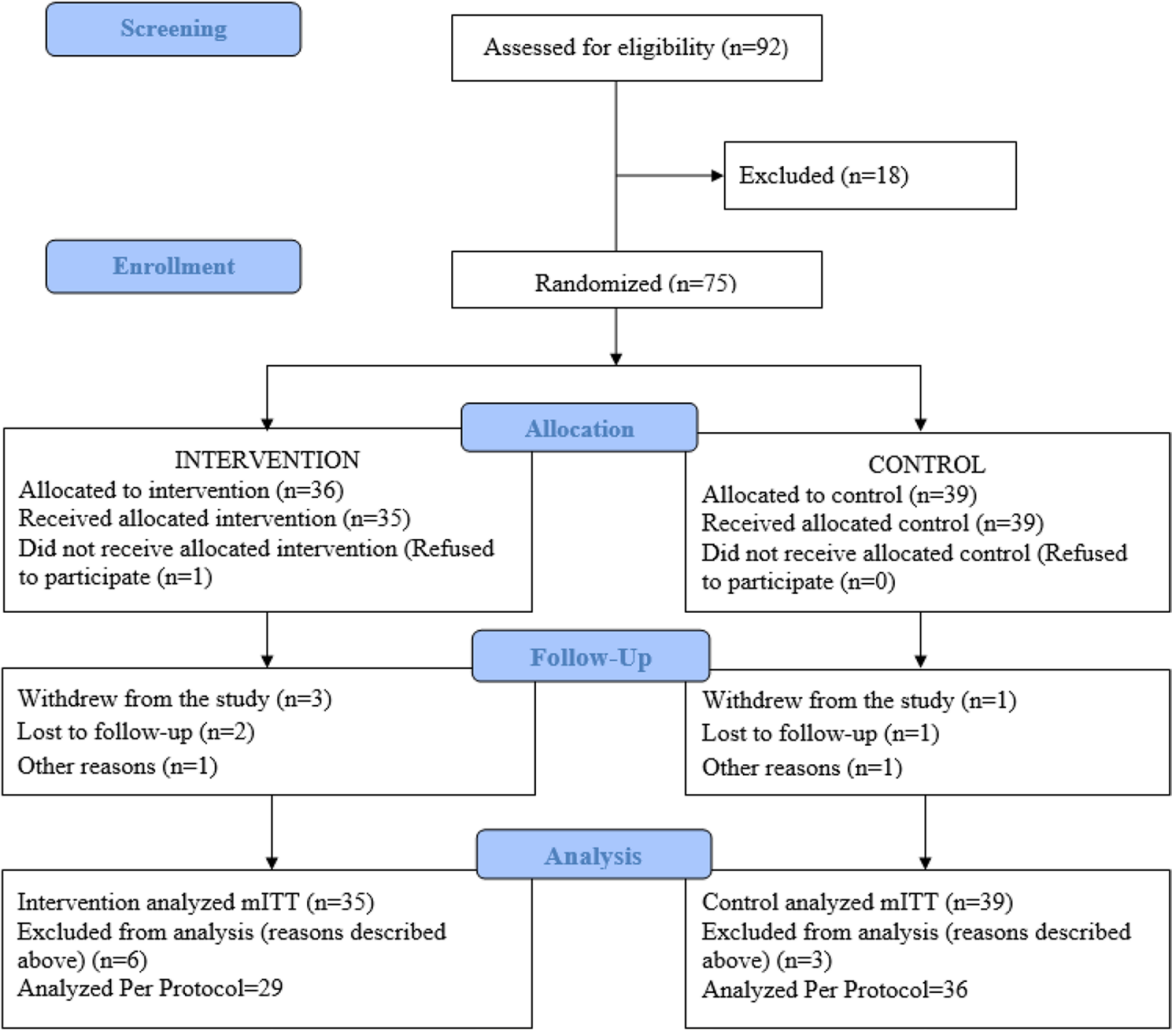
CONSORT flow diagram

Ninety-two subjects were screened according to the eligibility criteria described in Sect.  3.3. Seventy-five of them were randomized into two groups: CN2 (*n* = 36) and PBO (*n* = 39), from which 74 received study intervention or control and were defined as the mITT. Per protocol, a total of 65 participants completed the study as shown in Fig. 1. Participants were recruited between March, 2022 and May, 2023. Follow-up continued until November, 2023. Table 2 summarizes the demographics and baseline characteristics of the mITT population, with no significant differences between groups. The participants were mostly females (64.5%) with a mean age of 48.4 ± 11 years (CN2) and 49,2 ± 9,8 years (PBO) and comparable BMI of 24.2 kg/m^2^.

**Table 2:** Demographic and baseline data of the participants

Characteristics	CN2	PBO	*p*-value
Total number of subjects	35	39	
Number of males/females	10/25	16/23	0,2625^1^
Age (years)	48,4 (11,0)	49,2 (9,8)	0,7491
Weight (kg)	67,2 (11,6)	69,3 (14,6)	0,6892
Height (m)	1,7 (0,1)	1,7 (0,1)	0,4032
BMI (kg/m2)	24,2 (3,4)	24,2 (3,2)	0,9282
VAS at rest (cm)	1,53 (1,62)	1,24 (1,50)	0,5216
VAS during activity (cm)	3,98 (0,68)	3,99 (0,67)	0,9049
KOOS Pain (score)	68,17 (2,93)	71,01 (2,77)	0,4827
KOOS Symptoms (score)	48,06 (2,13)	50,73 (2.02)	0,3634
KOOS ADL (score)	73,70 (3,02)	75,23 (2,86)	0,7134
KOOS Sport/Rec (score)	48,71 (4,58)	52,31 (4,33)	0,5694
KOOS QOL (Score)	50,71 (3,82)	51,92 (3,62)	0,8187

*CN2* collagen native type II, *PBO* placebo, *BMI* body mass index, *VAS* visual analogue scale, *KOOS* knee injury osteoarthritis outcome score, *ADL* activities of daily living, *QOL* quality of life.

^1^ T-test. All values are mean ± SE.

### Activity evaluation

The time to pain onset was comparable between groups for the three standardized exercises, but the time to recover from pain in the cycling in the air (CIA) test at day 180 remained constant (0.71 min ± 0.10 min) for the CN2 group, whereas there was a significant increase compared to baseline in the PBO group (1,17 min ± 0.09 min; *p* = 0.0010). A significant between-group difference at day 180 (0.46 min ± 0.17 min; *p* = 0.0013) was detected for the CIA test, but not for going up and down stairs (UDS), or standing in one leg (SOL) tests. No differences were observed in the 6 min walking test.

### Joint discomfort evaluation

Although the results were exploratory in nature due to the profile of the participants included, clinically significant improvements from baseline were detected in KOOS Pain, QoL and Sport/Rec for the CN2 group, and only in KOOS Sport/Rec for the PBO group (Table 3). Statistical differences from baseline (*p* < 0.05) were detected for all subscores in both groups at 180 days, but appeared earlier in the CN2 group than in PBO for KOOS Pain (day 120 vs. 180), Symptoms (at day 90 vs. 120) and Quality of Life (at day 90 vs. 180).

**Fig. 2:**
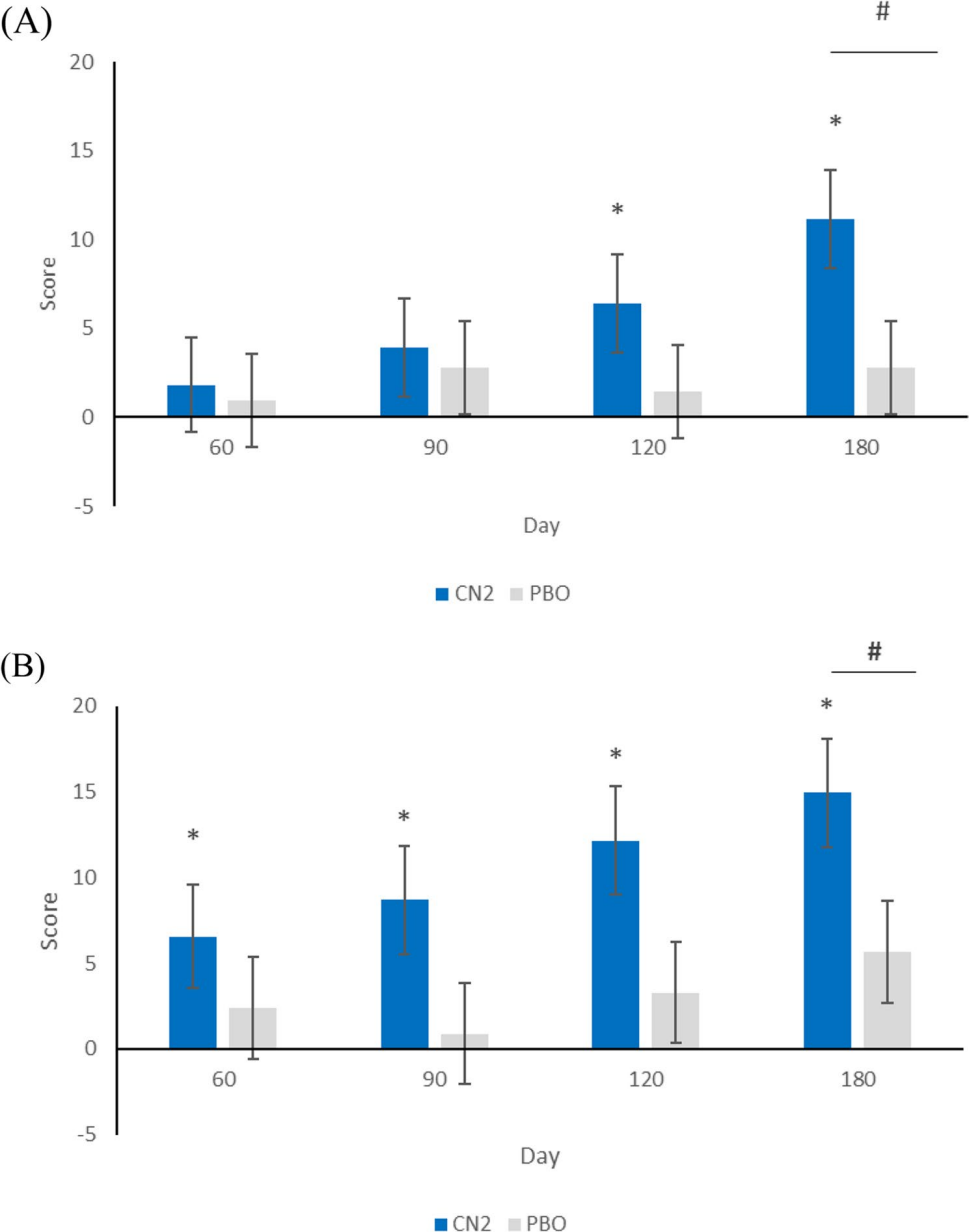
KOOS subscores of pain (**A**) and quality of life (**B**) in a subgroup of healthy individuals with a VAS score after exercise ≤4 cm at baseline. Results are expressed as difference in score from baseline after PBO or CN2 supplementation. *p <0.05 compared to baselines values #*p *<0.05 differences in changes from baseline values between groups. CN2, collagen native type II; PBO, placebo; KOOS, knee injury osteoarthritis outcome score

CN2 subjects with mild discomfort (VAS≤4 cm) during knee movement (*n* = 22) registered clinically significant improvements in in Sport/Rec (13.24 ± 4.22) at day 90, in Quality of Life at day 120 (12.16 ± 3.14) and in KOOS Pain (11.16 ± 2.77) and ADL (11.60 ± 2.94) at day 180, whereas no clinically significant improvements were registered in PBO subjects with mild discomfort during knee movement (*n* = 21). Statistical differences in improvement from baseline between groups were detected at day 180 in KOOS Pain (CN2 11.16 ± 2.77; PBO 2.78 ± 2.61; *p* < 0.0292) and KOOS Quality of Life (CN2 14.93 ± 3.14; PBO 5.65 ± 2.96; *p* < 0.0330) (Fig. 2). VAS value at rest and during knee movement had a significant reduction in both groups from day 60.

**Table 3:** Change in KOOS subscores in CN2 and PBO groups compared to baseline values

	Day	CN2	PBO
KOOS Pain	60	3.79 (± 2.58)	2.47 (± 2.38)
	90	3.89 (± 2.64)	3.18 (± 2.41)
	120	7.00 (± 2.67) *	4.53 (± 2.43)
	180	10.92 (± 2.67) *	8.23 (± 2.43)*
KOOS QoL	60	5.00 (± 2.796)	4.41 (± 2.57)
	90	8.32 (± 2.86) *	4.80 (± 2.60)
	120	11.44 (± 2.89)*	4.10 (± 2.62)
	180	14.46 (± 2.89)*	8.96 (± 2.62)*
KOOS Symptoms	60	3.94 (± 2.17)	3.06 (± 2.00)
	90	4.82 (± 2.22)*	3.83 (± 2.02)
	120	5.72 (± 2.24)*	4.34 (± 2.04)*
	180	9.29 (± 2.24)*	7.22 (± 2.04)*
KOOS ADL	60	3.99 (± 2.56)	3.41 (± 2.36)
	90	3.80 (± 2.62)	6.06 (± 2.38)*
	120	3.07 (± 2.65)	4.09 (± 2.40)
	180	9.10 (± 2.65)*	7.52 (± 2.40)*
KOOS Sport/Rec	60	4.30 (± 3.31)	4.16 (± 3.05)
	90	7.36 (± 3.39)*	8.18 (± 3.08)*
	120	5.93 (± 3.43)	6.97 (± 3.11)*
	180	13.35 (± 3.43)*	11.55 (± 3.11)*

*CN2* collagen native type II, *PBO* placebo, *KOOS* knee injury osteoarthritis outcome score, *ADL* activities of daily living, *QOL* quality of life, *VAS* visual analogue scale.

All values are mean ± SE.

*p value<0.05 compared to baseline.

### CTX-II concentration

**Fig. 3:**
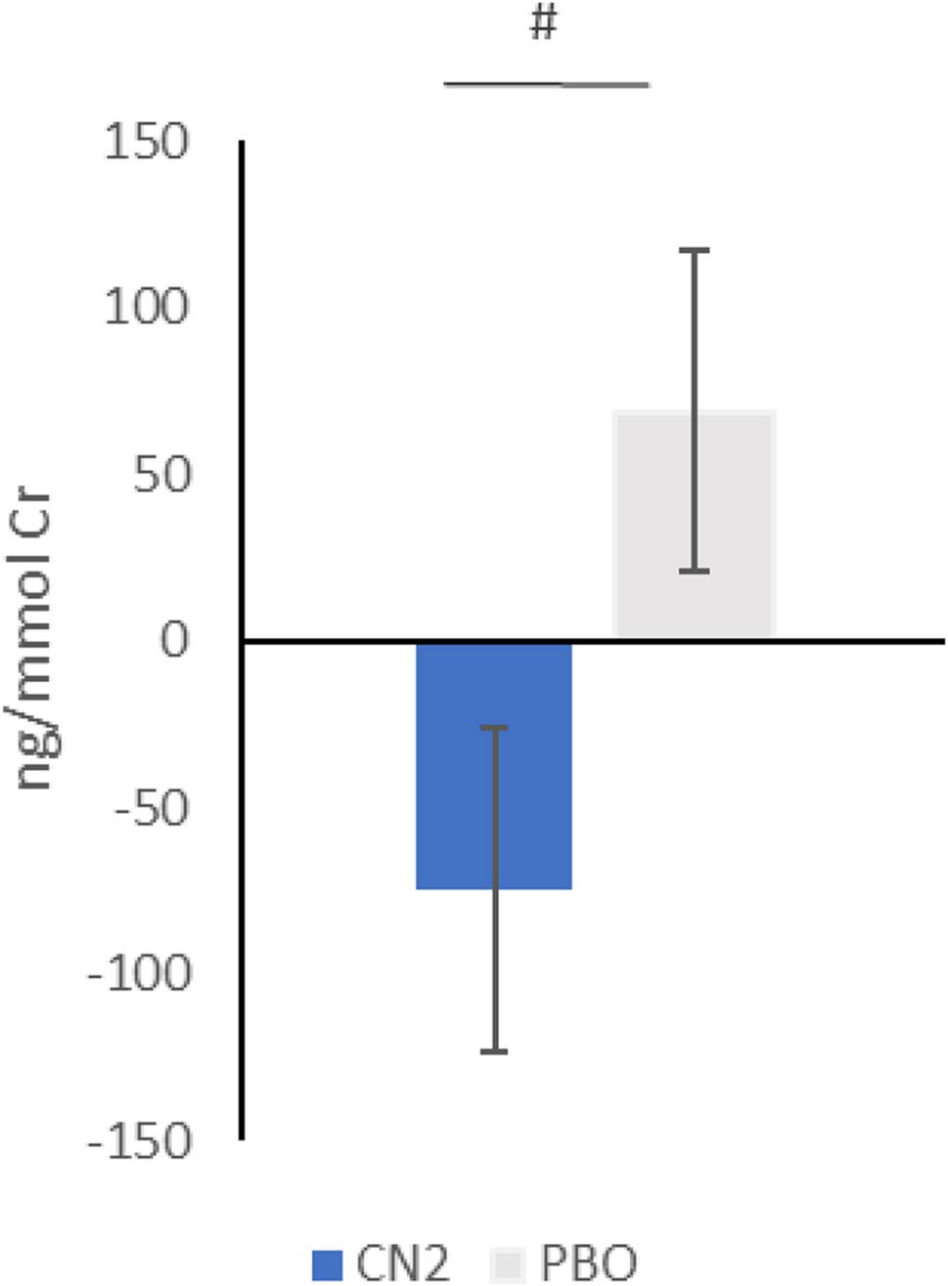
Change from baseline of the urinary CTX-II concentration in a subgroup of healthy individuals with a VAS score after exercise > 4 cm at baseline. Results are expressed as difference in concentration from baseline after PBO or CN2 supplementation. #*p *<0.05 differences in changes from baseline values between groups. CN2, collagen native type II; PBO, placebo; CTX-II, cross-linked C-terminal telopeptide of type II collagen

At the end of the study the urinary concentration of CTX-II decreased 5.7% (-19.87 ± 24.67 ng/mmol Cr) in the CN2 group and increased 7.4% (24.93 ± 22.54 ng/mmol Cr) in the PBO group as compared to baseline, but differences failed to reach statistical significance in the general population. However, a significant between-group difference (*p* = 0.0477) was obtained for the subgroup analysis with higher basal pain in movement (VAS>40 mm). In this subgroup CTXII decreased 18.3% (-74.63 ± 48.79 ng/mmol Cr) in the CN2 group (*n* = 13) while increased 20.6% (68.65 ± 48.08 ng/mmol Cr) in the PBO group (*n* = 18; Fig. 3).

### Safety assessment

No clinical or statistically significant changes were reported for any of the parameters assessed. No adverse events associated with the treatments were reported. No subjects reported taking rescue medication during the study period.

## Discussion

This study explored the impact of supplementation with CN2 on pain and joint function in healthy individuals who experienced joint discomfort as a result of an exercise protocol. Exercise induced joint pain has been shown to be frequently reported in the general population, particularly in amateur athletes [28], and it has been suggested to be a predisposing factor for the development of OA [29]. In fact, intensive joint loading exercise has been associated with cartilage damage [12, 13, 30] and synovial inflammation [17]. There is evidence supporting the role of the adaptative immune response in the initiation or amplification of inflammation at joint level [31, 32]. It has been suggested that cartilage damage results in the release of cartilage components that are recognized as autoantigens and are responsible for an oligoclonal T cell response [33]. Type II collagen is the main protein of joint cartilage and has been reported to be a potential source of autoantigens in different joint conditions [34–36]. Oral tolerance against type II collagen has been proven to induce cartilage protection, helping to control inflammation in different models of arthritis [21, 37–39]. There is also clinical evidence for the oral administration of low doses of native (undenatured) type II collagen in patients with OA [22, 40]. Although this immune-mediated mechanism of action has been described for OA and RA [21] we have explored whether potential benefit could be also detected in non-arthritic population reporting exercise-related joint discomfort.

In the present study, clinically significant improvements from baseline were found in non-osteoarthritic individuals supplemented with CN2 in KOOS pain, QoL and Sport/Rec. However, numerical improvements were also detected in the PBO group, and a clinically significant improvement was registered for the Sport/Rec subscale. Clinical improvements as a result of the PBO intervention have been extensively reported in OA studies [41]. In these studies, it has been estimated that up to 75% of the overall treatment effect could be attributable to contextual effects, commonly designated as placebo effect [42]. This is a limiting condition for OA studies, but could be even more limiting in studies with healthy populations [43]. For joint pain, a positive correlation has been clearly established between baseline pain and size of the placebo effect [44]. In this study, in order to reach statistical results mITT population with an effective size of 74 participants was selected and a preplanned sub-analysis was performed dividing the population into two groups based on the baseline value of pain as a result of the exercise loading activity. Interestingly, in the PBO group, clinically significant improvements were detected in the subgroup with moderate pain but not in the subgroup with mild pain. The lower placebo effect size in the subgroup of participants with lower baseline pain allowed the detection of statistical differences between groups in KOOS Pain and Quality of Life subscales. In general terms, it has been reported that KOOS Pain and QOL subscales are the most responsive to detect changes [26], which could explain why between-group differences were detected specifically in these sub-scores. The reduction of the placebo effect detected in the lower baseline pain subgroup is in agreement with previous findings from OA studies, which concluded that higher baseline pain results in greater placebo response [45].

Other studies have evaluated the potential benefit of native (undenatured) type II collagen supplementation in healthy subjects with exercise-associated joint discomfort [46–49]. All studies have reported improvements in activity-related joint discomfort and mobility, but differences exist in specific outcomes. For example, this study detected an improvement in time to pain recovery as compared to PBO after the CIA test, and some studies have reported improvements in time pain onset as a result of exercise [46, 49], while in this study no differences were detected in this parameter. In part, differences between studies could be related to the heterogeneity of the population recruited. It could be argued that the absence of a defined clinical condition in studies with healthy individuals reporting exercise-related joint discomfort, could potentially lead to a high interindividual response variability. In fact, previous studies using the KOOS questionnaire with this population profile have obtained mixed results [46, 49]. The heterogeneity of the population included is probably one potential limitation of this study. The inclusion of participants with unilateral or bilateral discomfort of variable duration has probably increased within-group dispersion. Another limitation of this study is the strong placebo effect in those participants with moderate basal pain during activity and the absence of multiplicity corrections. This dispersion added to the high placebo response may have limited the obtaining of between-group differences in some parameters.

The effect of native (undenatured) type II collagen supplementation in urinary CTX-II concentration has been previously explored in animal models [50–52] and in patients with OA [53], but to our knowledge, this is the first study in which it has been evaluated in healthy volunteers. Between-group differences were detected in the subgroup of participants with moderate basal pain in movement, suggesting a protective of effect of CN2 supplementation on cartilage, but failed to reach statistical significance in the general population. It has been reported that urinary CTX-II concentration is positively correlated with joint pain intensity [54, 55]. Taking into account the profile of the participants, this could explain the detection of differences in the subpopulation with higher basal pain but not in the general population.

Nevertheless, taken together, the overall results of this study suggest that oral supplementation with CN2 by healthy volunteers experiencing joint discomfort as a result of an exercise protocol over 6 months, is well tolerated and safe, and could help to improve joint discomfort, function and quality of life. Although further studies are warranted to confirm the clinical findings, these results add to a growing body of evidence showing the benefit of CN2 to support joint health in different conditions.

## Conclusion

This study found that supplementation with CN2, containing native (undenatured) type II collagen at a dose of 40 mg/d, is safe and provides symptomatic joint health benefits in people who experience milder pain after exercise, including improvements in joint function, discomfort and their impact on quality of life.

## Supplementary Information

Supplementary Material 1.

## Data Availability

The datasets generated and/or analysed during the current study are not publicly available due confidentiality and ethical reasons but are available from the corresponding author on reasonable request.

## Abbreviations

ADL: Activities of daily living
BMI: Body mass index
CIA: Cycling in the air
CN2: Collagen native type-II
CONSORT: Consolidated Standards of Reporting Trials
CTX-II: Cross-linked C-terminal telopeptide of type II collagen
DSP: Diastolic blood pressure
IL-1ꞵ: Interleukin 1-ꞵ
KOOS: Knee injury Osteoarthritis Outcome Score
MIA: Monoiodoacetate
mITT: Modified intention-to-treat population
NSAIDS: Non-steroidal anti-inflammatory drugs
OA: Osteoarthritis
PBO: Placebo
QOL: Quality of life
RA: Rheumatoid arthritis
SBP: Systolic blood pressure
SOL: Standing in one leg
SYSADOA: Symptomatic slow acting drugs for osteoarthritis
TNF𝛼: Tumor necrosis factor-𝛼
UDS: Up and down stairs
VAS: Visual analogue scale
YLD: Years lived with disability
